# *MiR-181b* suppresses proliferation of and reduces chemoresistance to temozolomide in U87 glioma stem cells^☆^

**DOI:** 10.1016/S1674-8301(10)60058-9

**Published:** 2010-11

**Authors:** Ping Li, Xiaoming Lu, Yingyi Wang, Lihua Sun, Chunfa Qian, Wei Yan, Ning Liu, Yongping You, Zhen Fu

**Affiliations:** a Department of Neurosurgery, the First Affiliated Hospital of Nanjing Medical University, Nanjing, Jiangsu 210029, China; b Department of Neurosurgery, the Affiliated Brain Hospital of Nanjing Medical University, Nanjing, Jiangsu 210029, China

**Keywords:** miR-181b, glioma stem cells, proliferation, chemoresistance

## Abstract

*MicroRNAs* regulate self renewal and differentiation of cancer stem cells. There, we sought to identify the expression of miR-181b in glioma stem cells and investigate the biological effect of miR-181b on glioma stem cells in this study. MiR-181b expression was measured by real-time PCR in glioma stem cells isolated from U87 cells by FACS sorting. After miR-181b was overexpressed in U87 glioma stem cells by miR-181b lentiviral expression vector and/or treatment of temozolomide, secondary neurosphere assay, soft agar colony assay and MTT assay were performed. Compared with U87 cells, the expression of miR-181b was significantly decreased in U87 glioma stem cells. Overexpression of miR-181b decreased neurosphere formation by U87 glioma stem cells *in vitro* and suppressed colony formation in soft agar, and the cell growth inhibition rates increased in a time-dependent manner in U87 glioma stem cells infected with miR-181b lentivirus. Furthermore, miR-181b had a synergistic effect on temozolomide-induced inhibition of secondary neurosphere and soft agar colony, and on cell growth inhibition rates. MiR-181b functions as a tumor suppressor that suppresses proliferation and reduces chemoresistance to temozolomide in glioma stem cells.

## INTRODUCTION

Accumulating evidence shows that tumor tissues contain cancer stem cells, which are the source of relapse and chemoresistance[1],[2]. The cancer stem cell model and hypothesis have greatly changed the basic science and clinical views of cancer. Recent studies have also reported that glioma stem cells can be identified in adult glioblastoma muleiforme (GBM)[3]–[5]. These glioma stem cells have several properties: self-renewal, formation of neurospheres, expression of genes associated with neural stem cells (NSCs), generation of daughter cells of different phenotypes from one mother cell, and differentiation into phenotypically diverse populations of cells that are similar to those present in the initial GBM. Because glioma stem cells have an extremely critical role in the initiation and recurrence of gliomas, studies focusing on glioma stem cells are being rapidly pursued. However, the molecular mechanism where by glioma stem cells involve in tumorgenesis is still unknown.

MicroRNAs (miRNAs) are single-stranded, noncoding RNAs of 20-22 nucleotides that regulate gene expression by binding to mRNA, leading to mRNA degradation or inhibition of translation. Abnormal expression of miRNAs has been linked to various disease processes, including cancer development and progression[6]. Recent data have suggested that miRNAs also have an essential role in self-renewal and the differentiation of cancer stem cells[7],[8]. In this study, we isolated glioma stem cells from cultured U87 cells and found that the expression of miR-181b was reduced in U87 glioma stem cells. Furthermore, miR-181b overexpression reduced the proliferation of glioma stem cells and sensitized them to temozolomide (TMZ). Our data indicate that miR-181b functions as a tumor suppressor in U87 glioma stem cells.

## MATERIALS AND METHODS

### Isolation and culturing of U87 glioma stem cells

Human glioma cell line U87 was purchased from the Chinese Academy of Sciences Cell Bank. U87 cells were maintained at 37°C in a 5% CO_2_ incubator in DMEM supplemented with 10% fetal bovine serum (FBS) and were routinely passaged at two- to three-day intervals. CD133 is a recognized marker for glioma stem cells. In our study, U87 glioma stem cells stained with allophycocyanin (APC)-conjugated CD133 antibodies (Miltenyi Biotec, USA) were sorted by fluorescence-activated cell sorting (FACS) on a FACS Calibur instrument. CD133-positive cells were designated as U87 glioma stem cells. These cells were then grown in DMEM/F12 medium (Gibco, USA) supplemented with B27 (Gibco, USA), recombinant human epidermal growth factor (rhEGF, 20 ng/mL; PeproTech, UK) and basic fibroblast growth factor (bFGF, 20 ng/mL; PeproTech, UK) at 37°C in an incubator 5% CO_2_ containing.

### Real-time quantification of miRNAs by stem-loop RT-PCR

Real-time quantification of miRNAs by stem-loop RT-PCR was described in our previous study[9]. Briefly, for the *Taq*Man-based real-time reverse transcription-polymerase chain reaction (RT-PCR) assays, the ABI 7300 HT Sequence Detection system (Applied Biosystem, USA) was used. All the primers for hsa-miR-181b (P/N: 4373116) and RNU6B endogenous controls (P/N: 4373381) for the *Taq*Man miRNA assays were purchased from Applied Biosystems (USA). Relative gene expression was calculated using the 2^−ΔΔCt^ method.

### Immunofluoresence staining to detect the expression of cell surface markers

Isolated CD133^+^ cells were cultured in serum-free medium to allow tumor spheres to form for one week. Neurospheres were fixed with 4% paraformaldehyde, permeabilized with 0.1% Triton X-100 and probed with mouse monoclonal antibodies against human CD133, nestin and GFAP (Abcam, UK). The nuclei of neurosphere cells were counterstained with DAPI. The surface markers listed above were detected and photographed with a laser scanning confocal microscope.

### Lentiviral vector construction and infection

A lentivirus-based vector for miR-181b was constructed with technical support from Shanghai GeneChem. Pre-miR-181b was annealed and inserted into the pGCSIL-GFP expression vector containing the U6 and CMV promoters. The miR-181b expression vector (pGC-LV) and packaging vectors (pHelper 1.0 and pHelper 2.0) were cotransfected into 293FT cells with Lipofectamine 2000 (Invitrogen, USA). The culture supernatants were collected, concentrated, and stored in a -70°C freezer. U87 glioma stem cells were infected with lentiviral vectors at a multiplicity of infection (MOI) of 100 in the presence of polybrene (10 µg/mL).

### Secondary sphere formation assays

The primary spheres were dissociated and mixed with miR-181b lentivirus (MOI=100) for 8 h. Then, 200 cells were dispensed into each well of a 96-well plate in 0.2 mL of growth media. The secondary sphere numbers were counted after 7 d in culture.

### Soft agar colony assay

Soft agar analysis was performed as described in our previously published paper[9]. Briefly, the primary spheres were dissociated and mixed with the miR-181b lentivirus (MOI=100) for 8 h. Then, 1×10^4^ cells were seeded in 0.35% agar in the middle of the agar. In each well of a 24-well plate, 0.5% agar was plated at the bottom, and 0.35% agar was plated at the top. Plates were incubated in a 37°C, 5% CO_2_ incubator for 2 w. Colonies were photographed and counted after incubation.

### Western blotting assay

To determine the levels of protein expression, total protein was isolated in lysis buffer (137 mmol/L NaCl, 15 mM EGTA, 100 µmol/L phenylmethylsulfonyl fluoride, 0.1 mmol/L sodium orthovanadate, 15 mmol/L MgCl_2_, 0.1% Triton X-100, 25 mmol/L MOPS, and 20 µmol/L leupeptin, adjusted to pH 7.2). Equal amounts of protein (30 µg) were loaded onto the sample wells and were resolved on a 12% sodium dodecyl sulfate-polyacrylamide gel. The electrophoresed proteins were transferred to a polyvinylidene difluoride immobilon-P membrane (Millipore, Watford, UK) and were subjected to immunoblot analysis with approprate antibodies. Membranes were reblotted for anti-GAPDH antibodies to check for equal loading of samples. Anti-cyclin D1, c-Myc and Ki-67 antibodies were purchased from Santa Cruz Biotechnology (USA). Anti-GAPDH antibody was obtained from KangChen Bio-tech Inc., Shanghai.

### Cytotoxicity assays

The cytotoxic effect of TMZ and miR-181b on U87 glioma stem cells was determined by MTT assay. U87 glioma stem cells were plated at 1×10^3^ cells per well in 96-well plates with six replicated wells for each condition and infected with lentivirus for 24 h. Then, TMZ (100 µmol/L, Sigma, USA) was added and the cells were incubated and assayed for a further 24, 48, 72, 96 and 120 h. MTT solution was added to each well and incubated at 37°C for 2 h. The reaction was stopped by adding dimethyl sulfoxide. Cell viability was determined at 540 nm absorbance via a scanning multiwell spectrophotometer. All data points represent the mean of a minimum of six wells. The cell growth inhibition rate was calculated by the formula (AC-AT)/AC×100% (AC=absorbance value of the control group; AT=absorbance value of the experimental group).

### Statistical analysis

All statistical analysis was performed using SPSS 11.0. Descriptive statistics included the mean and SE, and one-way ANOVA and *t*-test were used to determine significant differences between groups. *P* < 0.05 was considered to be statistically significant.

## RESULTS

### Isolation and characterization of cancer stem cells isolated from U87 cells

**Fig. 1:**
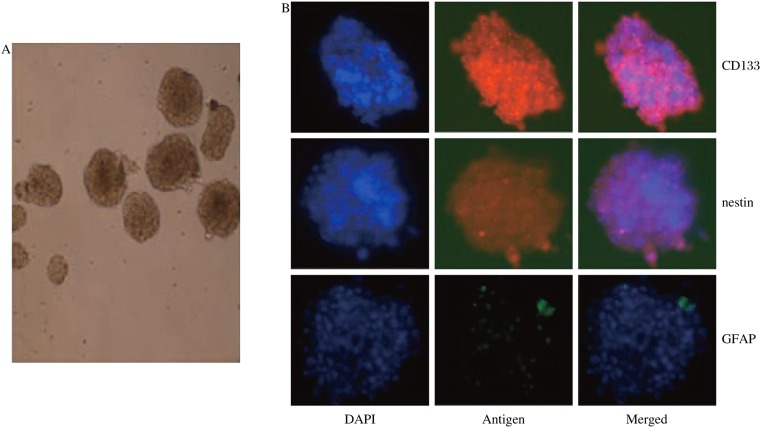
Characterization of U87 glioma stem cells. A: U87 glioma stem cells formed significant spheres in serum-free media supplemented with fibroblast growth factor and epidermal growth factor. B: The spheres were stained with anti-CD133, nestin, and GFAP antibodies.

After staining with anti-CD133 antibody, CD133-positive U87 cells were selected via FACS sorting. CD133-positive cells were then cultured in serum-free DMEM/F12 media supplemented with EGF, FGF, and B27 for 1 w. Then, an inverted microscope was used to observe the formation of neurospheres, and the spheres were photographed (***Fig.1A***). Neurospheres were stained for the stem cell markers CD133 and nestin and the differentiation marker GFAP. DAPI was used for nuclear staining (***Fig.1B***). The data showed that glioma stem cells were successfully isolated from U87 cell cultures.

### MiR-181b is down-regulated in U87 glioma stem cells

**Fig. 2:**
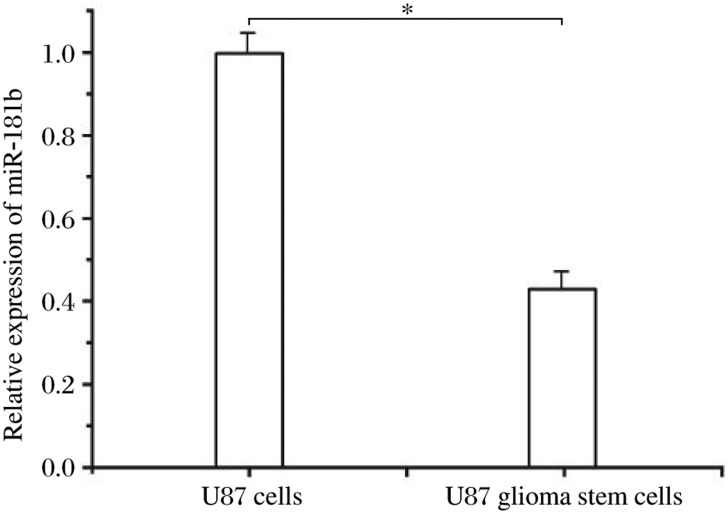
MiR-181b is reduced in U87 glioma stem cells compared with normal U87 cells. Real-time PCR was used to analyze the expression of miR-181b in U87 glioma stem cells. Compared with normal U87 cells, *P < 0.05.

To examine the expression of miR-181b in U87 glioma stem cells, we carried out real-time PCR to analyze the expression of miR-181b in CD133-positive and normal U87 cells. As shown in ***Fig. 2***, the expression of miR-181b in normal U87 cells was 2.3-fold higher than that in U87 glioma stem cells, suggesting that miR-181b may play an important role in U87 glioma stem cells.

### MiR-181b suppresses the proliferation of U87 glioma stem cells *in vitro*

**Fig. 3:**
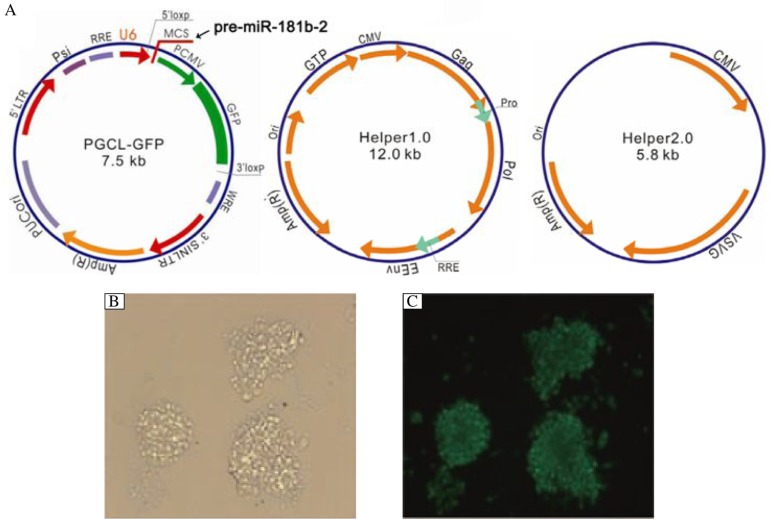
MiR-181b lentiviral expression vectors. A: The structures of three plasmids used in the construction of the miR-181b lentiviral expression vector. B: At d 7 postinfection, the efficiency of infection was monitored by detecting GFP expression using phase-contrast microswpy. C: The same field observed by fluorescence microscopy.

**Fig. 4:**
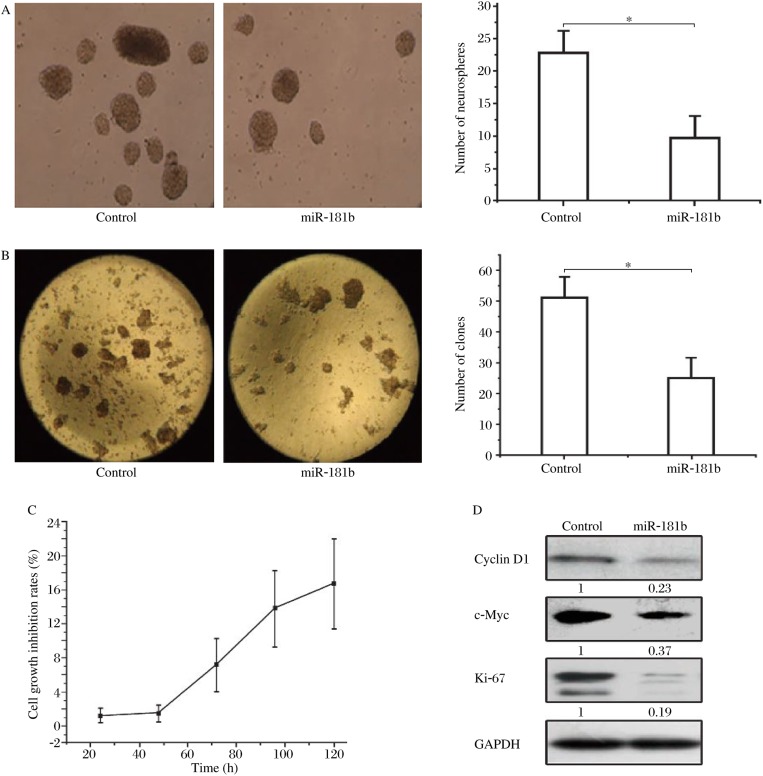
MiR-181b inhibits U87 glioma stem cell proliferation *in vitro*. A: MiR-181b decreased neurosphere formation by U87 glioma stem cells *in vitro*. B: MiR-181b suppressed colony formation in soft agar. C: Cell growth inhibition rates increased in a time-dependent manner in glioma stem cells infected with miR-181b lentivirus. D: miR-181b reduced the expression of proliferation-related proteins such as cyclin D1, c-Myc and Ki-67. Compared with controls, *P < 0.05.

To determine whether miR-181b has an effect on the proliferation of U87 glioma stem cells, we first successfully constructed pre-hsa-miR-181b lentiviral expression vectors (***Fig. 3***). Then, a secondary neurosphere assay and soft agar colony assay were used. As shown in ***Fig. 4A*** and ***4B***, overexpression of miR-181b decreased the sphere formation of U87 glioma stem cells *in vitro* and suppressed colony formation in soft agar. The cell growth inhibition rates increased in a time-dependent manner in U87 glioma stem cells infected with miR-181b lentivirus (***Fig. 4C***). In additiion, miR-181b overexpression reduced the expression of proteins associated with proliferation, such as cyclin D1, c-Myc and Ki-67 (***Fig. 4D***).

### miR-181b sensitize U87 glioma stem cells to TMZ

**Fig. 5:**
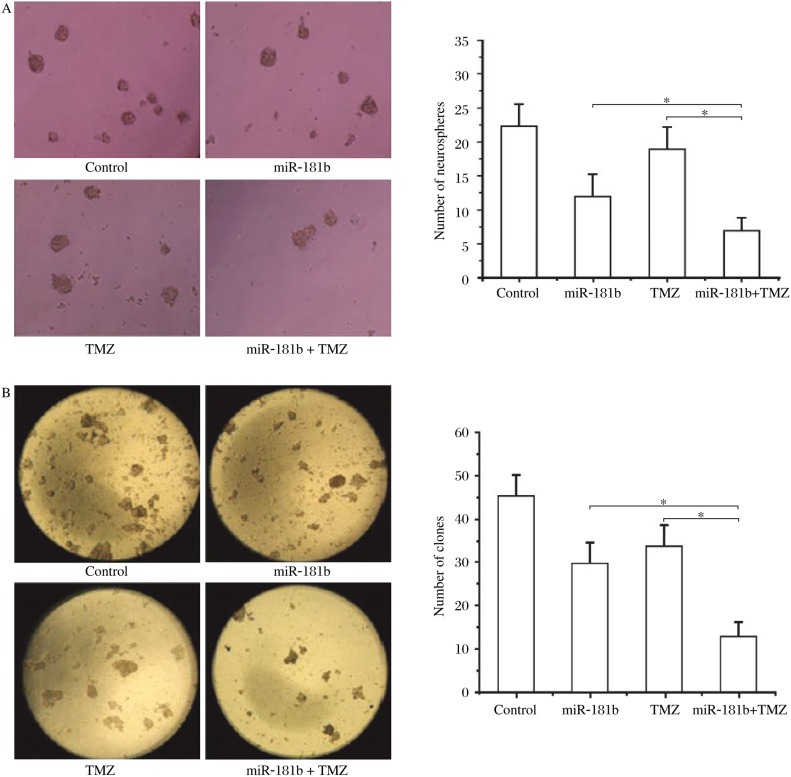
MiR-181b enhances TMZ-induced inhibition of neurosphere and soft agar colony formation. A: MiR-181b had a synergistic effect on the TMZ-induced inhibition of secondary neurosphere formation in glioma stem cells. B: MiR-181b had a synergistic effect on the TMZ-induced inhibition of soft agar colony formation in glioma stem cells. Compared with TMZ and miR-181b groups, *P < 0.05. TMZ: temozolomide.

**Fig. 6:**
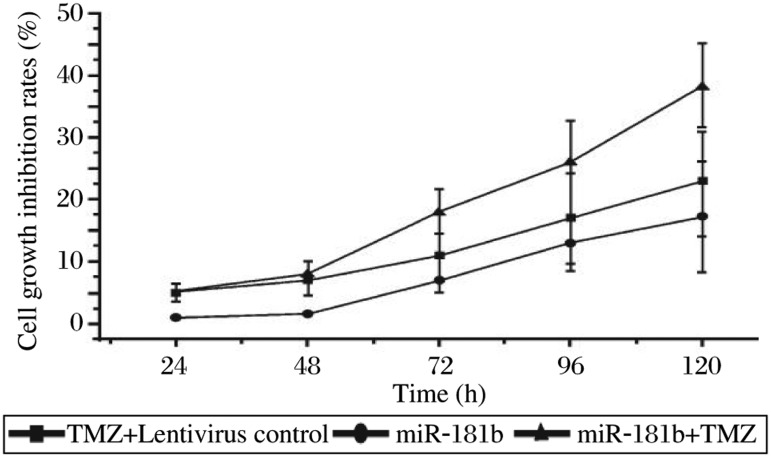
miR-181b enhances TMZ-induced growth inhibition. Cells treated with miR-181b and TMZ (100 µmol/L) from 24-120 h were subjected to the MTT assays. The viability of control cells was considered 100%. Compared with TMZ and miR-181b groups, *P < 0.05. TMZ: temozolomide.

It has been reported that cancer stem cells contribute to chemoresistance in various tumors. In this study, we examined whether miR-181b sensitizes U87 glioma stem cells to TMZ. As shown in ***Fig. 5A*** and ***Fig. 5B***, miR-181b had a synergistic effect on TMZ-induced inhibition of formation of secondary neurospheres and soft agar colonies by U87 glioma stem cells. The cell growth inhibition rates increased in a time-dependent manner in U87 glioma stem cells treated with TMZ and miR-181b lentivirus (***Fig. 6***). The cytotoxicity induced by TMZ in U87 glioma stem cells significantly increased 72 h after infection with miR-181b lentivirus. These results suggest that upregulation of miR-181b can sensitize U87 glioma stem cells to TMZ-induced growth inhibition.

## DISCUSSION

In the past few years, cancer stem cells have been identified in gliomas. Glioma stem cells are responsible for the recurrence as well as the chemo- and radio-resistance of gliomas[3]–[5]. In this study, we showd that miR-181b was down-regulated in U87 glioma stem cells. Overexpression of miR-181b suppressed the proliferation of U87 glioma stem cells. Furthermore, miR-181b overexpression sensitized U87 glioma stem cells to TMZ-induced growth inhibition.

It has been well-documented that miRNAs function as tumor suppressors or promoters by regulating the expression of signaling molecules, such as cytokines, growth factors, transcription factors, and proapoptotic and antiapoptotic genes in different cancer[9]–[12]. Recent studies have shown that some miRNAs are aberrantly expressed in cancer stem cells[13]–[16]. However, the expression profile of miRNAs in glioma stem cells has not been fully addressed. Our previous study showed that miR-125b was down-regulated in U251 glioma stem cells[17]. Here, we analyzed U87 glioma stem cells and normal U87 cells to identify whether the expression of miR-181b was significantly different between the two groups. Our results showed that miR-181b expression was significantly lower in U87 glioma stem cells.

Glioma stem cells are characterized by self-renewal, limitless proliferation, tumor initiation, multi-potent differentiation and expression of stem cell surface markers such as CD133 and nestin. Their unlimited proliferative potential is a key factor in tumor development and maintenance[18],[19]. It has been reported that miR-124 and miR-137 induce the differentiation of brain tumor stem cells. Our previous study showed that miR-125b is critical for the suppression of human U251 glioma stem cell proliferation through regulating the cell cycle proteins CDK6 and CDC25A[17]. In this study, we identified the biological effects of miR-181b on U87 glioma stem cells by infecting these cells with a lentiviral miR-181b vector. Overexpression of miR-181b consistently suppressed the proliferation of U87 glioma stem cells. These data indicate that one or more processes related to the self-renewal ability of U87 glioma stem cells is affected by miR-181b, indicating that miR-181b reduces stemness in U87 glioma stem cells. To the best of our knowledge, this study is the first to demonstrate that miR-181b is a tumor suppressor in U87 glioma stem cells.

Accumulating data indicate that aberrant expression of miRNAs is involved in the chemoresistance of cancers[20]. MiR-215 leads to an increase in chemoresistance to MTX and TDX through the suppression of DTL expression in osteosarcoma and colon cancer cells[21]. MiR-21 targets LRRFIP1 and contributes to VM-26 resistance in GBM[22]. In addition, reduction of miR-21 increases the chemosensitivity of glioma cells to taxol[23]. Meanwhile, GBM stem cells have a high resistance to chemotherapeutic drugs[24]. A recent study has shown that cancer stem cells from a human glioma cell line are resistant to Fas-induced apoptosis[25]. In the present study, we showed that miR-181b overexpression sensitized U87 glioma stem cells to TMZ-induced growth inhibition. It has been reported that miR-181b modulates multidrug resistance in human cancer cell lines by targeting Bcl-2, a protein that has an important anti-apoptotic role[26]. Thus, it is possible that miR-181b may modulate the chemoresistance of glioma stem cells by targeting Bcl-2. However, the mechanism of miR-181b involvement in glioma stem cell chemoresistance is still unclear and warrants further investigation.

In summary, this study highlights the importance of miR-181b in regulating the stemness of glioma stem cells. Overexpression of miR-181b suppresses proliferation and reduces chemoresistance to TMZ in U87 glioma stem cells. These data suggest that miR-181b could potentially serve as a therapeutic agent for glioma by eradicating glioma stem cells.

## References

[1] Hermann PC, Bhaskar S, Cioffi M, Heeschen C (2010). Cancer stem cells in solid tumors. Semin Cancer Biol.

[2] Kruyt FA, Schuringa JJ (2010). Apoptosis and cancer stem cells: Implications for apoptosis targeted therapy. Biochem Pharmacol.

[3] Yuan X, Curtin J, Xiong Y, Liu G, Waschsmann-Hogiu S, Farkas DL (2004). Isolation of cancer stem cells from adult glioblastoma multiforme. Oncogene.

[4] Galli R, Binda E, Orfanelli U, Cipelletti B, Gritti A, De Vitis S (2004). Isolation and characterization of tumorigenic, stem-like neural precursors from human glioblastoma. Cancer Res.

[5] Altaner C (2008). Glioblastoma and stem cells. Neoplasma.

[6] Garzon R, Fabbri M, Cimmino A, Calin GA, Croce CM (2006). MicroRNA expression and function in cancer. Trends Mol Med.

[7] DeSano JT, Xu L (2009). MicroRNA regulation of cancer stem cells and therapeutic implications. AAPS J.

[8] Ji Q, Karnak D, Hao P, Wang R, Xu L (2010). No small matter: microRNAs - key regulators of cancer stem cells. Int J Clin Exp Med.

[9] Shi L, Cheng Z, Zhang J, Li R, Zhao P, Fu Z (2008). Hsa-mir-181a and hsa-mir-181b function as tumor suppressors in human glioma cells. Brain Res.

[10] Zhou X, Ren Y, Moore L, Mei M, You YP, Xu P (2010). Downregulation of miR-21 inhibits EGFR pathway and suppresses the growth of human glioblastoma cells independent of PTEN status. Lab Invest.

[11] Esquela-Kerscher A, Slack FJ (2006). Oncomirs - microRNAs with a role in cancer. Nat Rev Cancer.

[12] Davalos V, Esteller M (2010). MicroRNAs and cancer epigenetics: a macrorevolution. Curr Opin Oncol.

[13] Yu F, Yao H, Zhu P, Zhang X, Pan Q, Gong C (2007). let-7 regulates self renewal and tumorigenicity of breast cancer cells. Cell.

[14] Ji Q, Hao X, Zhang M, Tang W, Yang M, Li L (2009). MicroRNA miR-34 inhibits human pancreatic cancer tumor-initiating cells. PLoS One.

[15] Yu F, Deng H, Yao H, Liu Q, Su F, Song E (2010). Mir-30 reduction maintains self-renewal and inhibits apoptosis in breast tumor-initiating cells. Oncogene.

[16] Garzia L, Andolfo I, Cusanelli E, Marino N, Petrosino G, De Martino D (2009). MicroRNA-199b-5p impairs cancer stem cells through negative regulation of HES1 in medulloblastoma. PLoS One.

[17] Shi L, Zhang J, Pan T, Zhou J, Gong W, Liu N (2010). MiR-125b is critical for the suppression of human U251 glioma stem cell proliferation. Brain Res.

[18] Flores DG, Ledur PF, Abujamra AL, Brunetto AL, Schwartsmann G, Lenz G (2009). Cancer stem cells and the biology of brain tumors. Curr Stem Cell Res Ther.

[19] Singh SK, Clarke ID, Hide T, Dirks PB (2004). Cancer stem cells in nervous system tumors. Oncogene.

[20] Iorio MV, Croce CM (2009). MicroRNAs in cancer: small molecules with a huge impact. J Clin Oncol.

[21] Song B, Wang Y, Titmus MA, Botchkina G, Formentini A, Kornmann M (2010). Molecular mechanism of chemoresistance by miR-215 in osteosarcoma and colon cancer cells. Mol Cancer.

[22] Li Y, Li W, Yang Y, Lu Y, He C, Hu G (2009). MicroRNA-21 targets LRRFIP1 and contributes to VM-26 resistance in glioblastoma multiforme. Brain Res.

[23] Ren Y, Zhou X, Mei M, Yuan XB, Han L, Wang GX (2010). MicroRNA-21 inhibitor sensitizes human glioblastoma cells U251 (PTEN-mutant) and LN229 (PTEN-wild type) to taxol.. BMC Cancer.

[24] Eramo A, Ricci-Vitiani L, Zeuner A, Pallini R, Lotti F, Sette G (2006). Chemotherapy resistance of glioblastoma stem cells. Cell Death Differ.

[25] Bertrand J, Begaud-Grimaud G, Bessette B, Verdier M, Battu S, Jauberteau MO (2009). Cancer stem cells from human glioma cell line are resistant to Fas-induced apoptosis. Int J Oncol.

[26] Zhu W, Shan X, Wang T, Shu Y, Liu P (2010). miR-181b modulates multidrug resistance by targeting BCL2 in human cancer cell lines. Int J Cancer.

